# Physical Property Evolution of the Anode Mixture during the Baking Process

**DOI:** 10.3390/ma14040923

**Published:** 2021-02-15

**Authors:** Bowen Chen, Hicham Chaouki, Donald Picard, Julien Lauzon-Gauthier, Houshang Alamdari, Mario Fafard

**Affiliations:** 1 NSERC/Alcoa Industrial Research Chair MACE3 and Aluminium Research Centre-REGAL, Department of Civil and Water Engineering, Université Laval, Quebec, QC G1V 0A6, Canada; hicham.chaouki@gci.ulaval.ca (H.C.); mario.fafard.2@ulaval.ca (M.F.); 2 Eddify Technologies Company, Quebec, QC G1P 0B3, Canada; dpicard@eddyfi.com; 3 Continuous Improvement Smelting Technology, Alcoa, Deschambault-Grondines, QC G0A 1S0, Canada; julien.lauzon-gauthier@alcoa.com; 4 NSERC/Alcoa Industrial Research Chair MACE3 and Aluminium Research Centre-REGAL, Department of Mining, Metallurgical and Materials Engineering, Université Laval, Quebec, QC G1V 0A6, Canada; houshang.alamdari@gmn.ulaval.ca

**Keywords:** Hall-Héroult process, prebaked anode, thermogravimetric analysis, dilatometry, permeability, real density, porosities, shrinking index, pore pressure

## Abstract

The Hall-Héroult process uses prebaked carbon anodes as electrodes. The anode’s quality plays a crucial role in the efficiency of the aluminium production process. During the baking process, the anode undergoes complex physicochemical transformations. Thus, the production of high-quality anodes depends, among others, on the efficient control of their baking process. This paper aims to investigate the evolution of some physical properties of the anode paste mixture during the baking process. These properties include the mass loss fraction, real and apparent densities, the ratio of apparent volume, the permeability, and porosities. For this purpose, experiments consisting of thermogravimetric analysis, dilatometry, air permeability, and helium-pycnometric measurements were carried out. The anode permeability at high temperatures was linked to the air permeability through a permeability correlator due to experimental limitations. Moreover, the real density at high temperatures was estimated by combining real densities of the coal tar pitch and coke aggregates. Different porosities, such as the open porosity and the closed porosity related to the pitch binder, were estimated by taking the permeability at high temperatures into account. In this context, the effect of the permeability correlator, which was introduced to link the permeability at high temperatures to the air permeability, was investigated through a sensitivity analysis. These results allow an estimation of the shrinking index, a new variable introduced to reflect the baking level of the anode mixture, which is linked to the volatile that is released in both open and closed pores. Afterwards, the pore pressure inside closed pores in the coal tar pitch was estimated. The obtained results highlight some new insights related to the baking process of the anode mixture. Moreover, they pave the way for better modeling of the thermo-chemo-mechanical behavior of anodes at high temperatures.

## 1. Introduction

Carbon anodes are utilized as positive electrodes in the Hall-Héroult electrolysis process for the production of primary aluminium. Søderberg anodes and prebaked anodes are two basic designs in anode technology. At present, the prebaked anode technology is often adopted in the aluminium production industry, because of its advantage in increasing the energy efficiency and reducing carbon consumption and emission of hazardous gases. The prebaked anodes are highly consumable when compared with the other industrial carbonaceous materials utilized in the electrolysis cell [1]. Thus, high-quality anodes are required to maintain the sustainability of the operation, which substantially increases the efficiency of the electrolysis process. One of the most important solutions could be the control and improvement of the anode quality via its production process [2].

In the anode production industry, the anode baking is considered to be the most cost-intensive stage as well as the most frequent cause of anode problems [3]. Being formed by vibro-compaction/compaction process [4], a green anode mixture consists of carbon mixtures and pores. The carbon mixtures are typically composed of petroleum coke aggregates (≈65 wt.%), coal tar pitch (≈15 wt.%), and recycled anode butts (≈20 wt.%) [5]. The baking process transforms a green anode into a prebaked anode. During this process, the pitch carbonization binds up the coke aggregates and discharges the light binder volatile into the pore space, such that a solid-gas anode mixture is formed. The pressure built-up in pores entrapping the volatile will cause a swelling of the apparent volume [6], facilitating the pitch carbonization [7]. The volume shrinkage due to the carbonization process will deeply modify the microcrystalline structure of carbon mixtures and porous networks in the anode [8,9]. Therefore, the prevalence of macrocracks will result at the end of the baking process.

The physiochemical transformation effect on the properties of the anode mixture is still not fully understood. During the baking process, a physical property of the anode material is mostly expressed as a function of the temperature. However, the anode properties are unable to recover their initial state after the baking process, owing to the chemical reaction of the pitch pyrolysis. Many authors have attempted to propose variables that reflect the baking level of the anode or the ramming paste [3,10,11,12]. Nevertheless, these attempts lack consideration for the impacts of the chemical pyrolysis and the pore pressure on the anode properties. For this reason, a new internal state variable, called the “shrinking index”, was introduced in this work to include the pitch pyrolysis on modelling the baking process [13].

Carbon anodes contain the pores that are either inherent in the microstructure of raw materials or formed in the manufacturing process [14]. During the baking process, the porosity in the anode has much impact on the anode volume [3,6], and the porosity in the pitch has been found to significantly develop with the weight loss due to the pitch pyrolysis [15]. At high temperatures, before reaching the maximum expansion, pitch-bonded carbon materials will swell while losing mass [16,17,18]. In [19], the weight of the coal tar pitch was used to quantitatively analyze the kinetics of pitch pyrolysis, and the thermogravimetric analysis (TGA) was, for the first time, adopted in order to investigate the thermal decomposition of the pitch. In [20,21,22], the investigations of the pyrolysis process in carbon electrodes were reported and the obtained results were used to identify parameters of a power-law model, such as the activation energy and the pre-exponential factor. By exceeding the maximum expansion, the volume of a pyrolyzing carbon material shrinks up by decreasing the pore volume and releasing the entrapped volatile in bubble closed pores into the open crack pores formed by the shrinking of the pitch binder [9]. In this condition, the anode permeability influences diffusion velocity describing the volatile transportation in the anode [23]. In this regard, a Kozeny model linking the open porosity with the anode permeability was used to simulate the gas pressure in the anode during baking [24,25].

This work aims to investigate the evolution of relevant physical properties of the anode paste mixture during the baking process. These properties include the mass loss fraction, real and apparent densities, the ratio of apparent volume, the permeability, and porosities. For this purpose, four main experimental characterizations were achieved in this work: (1) thermogravimetric analysis (TGA) to obtain the mass loss of the anode at high temperatures due to the pitch pyrolysis; (2) helium-pycnometric measurement to determine the real pycnometric density of the anode paste at room temperature; (3) air permeability measurement of the anodes that were baked up to different high temperatures to estimate the open porosity at high temperatures; and, (4) dilatometry to obtain the ratio of apparent volume at high temperatures. To this end, lab-scale anode samples were fabricated while using the servohydraulic compaction method [26]. Moreover, the real density at high temperatures was estimated by combining real densities of the coal tar pitch and coke aggregates. The anode permeability at high temperatures was linked to the air permeability through a permeability correlator due to experimental limitations. Different porosities, such as the open porosity and the closed porosity related to the pitch binder, were estimated by taking the permeability at high temperatures into account. In this context, the effect of the permeability correlator, introduced to link the permeability at high temperatures to the air permeability, was investigated through a sensitivity analysis. Based on the results, the shrinking index characterizing the baking process was obtained. In [27], the concentration of each component of the polycyclic aromatic hydrocarbons (PAHs) was chromatographically analyzed. Subsequently, the gas pressure in closed pores that are related to the pitch binder were further estimated.

## 2. Methodology

### 2.1. Preparation of Anode Samples

The raw materials, which were used to fabricate lab-scale green samples, were provided by the aluminium smelting plant Alcoa Deschambault (Deschambault, QC, Canada). The recipe consists of the calcined coke aggregates (86.06 wt.%) (≤8 US Mesh) and the coal tar pitch (13.94 wt.%). The size distribution of coke aggregates in the anode sample and the properties of the coal tar pitch are, respectively, tabulated in Table 1; Table 2.

**Figure 1:**
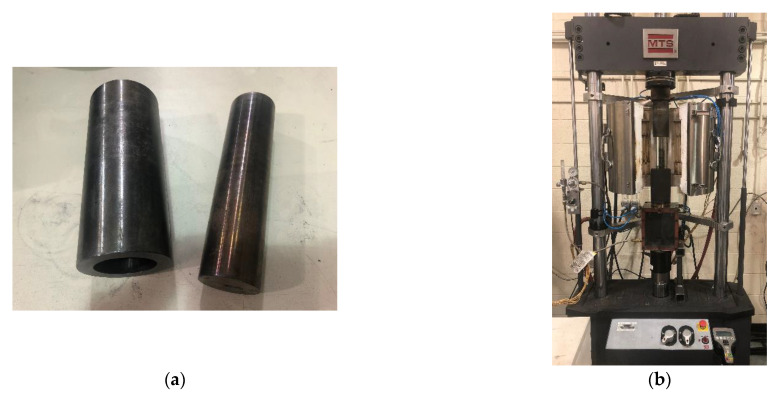
Device for the compaction of the anode paste. (**a**) Rigid die; (**b**) MTS Servohydraulic press.

The green samples, with dimensions of 50 mm × 100 mm (diameter × height), were fabricated while using a MTS Servohydraulic press (MTS, Eden Prairie, MN, USA), as shown in Figure 1. The compaction of the anode paste was performed at 150 °C under a maximum uniaxial pressure of 70 MPa [26]. These samples were divided into three groups. The samples in the first group were cut into ones having the dimensions 50 mm × 50 mm (diameter × height), and they were used for the thermogravimetric analysis (TGA) and the dilatometry. Those in the second group were cut into samples having the dimensions of 50 mm × 20 mm (diameter × height), and they were used for the air permeability measurement. The last group samples were used for the helium-pycnometric measurement.

**Figure 2:**
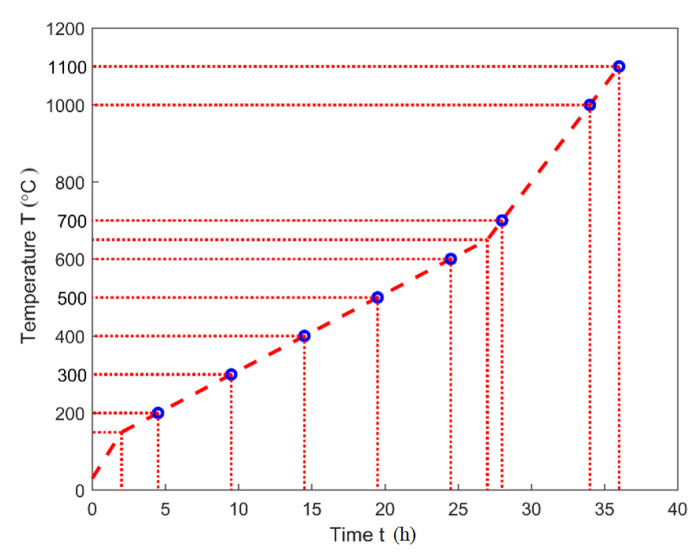
Heating program [28].

The heating program, as shown in Figure 2, was used for the TGA analysis, the dilatometry, the air permeability, and the helium-pycnometric measurements. The red dashed line corresponds to the heating program of the TGA analysis, where the samples were baked up to 1000 °C. Blue circles correspond to given target temperatures $T^{*}$. Samples were baked up to $T^{*}$ and then cooled down to the room temperature for the dilatometry ($T^{*}$ at 1100 °C), for the air permeability measurement ($T^{*}$ ranging from 200 °C to 1100 °C excluding 1000 °C) and for the helium-pycnometric measurement ($T^{*}$ ranging from 200 °C to 1000 °C). Table 3 categorizes the samples that aimed for different characterizations.

### 2.2. Thermogravimetric Analysis (TGA)

**Figure 3:**
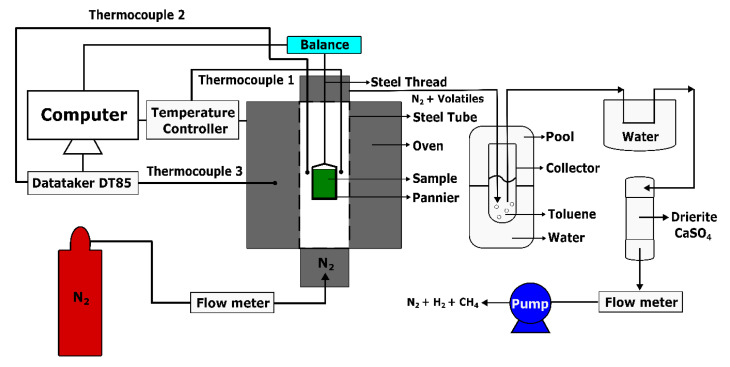
Schematic representation of Thermogravimetric Analysis (TGA) set-up.

The thermogravimetric analysis was intended to obtain the mass loss fraction of the anode at high temperatures for calculating the real and apparent densities of the anode in Section 3.2.1 and Section 3.2.2. This analysis was carried out using an experimental set-up, which was developed in the Aluminium Research Centre—REGAL of Laval University (Québec, QC, Canada), according to the international standard ISO 12988-2 [29], as shown in Figure 3. The set-up consists of an oven (ATS: Applied Test System) that can reach a temperature of 1200 °C. The oven enclosure contains a steel tube acting as a pyrolysis chamber, having a length of 1030 mm and a diameter of 100 mm. A pannier, which was used to hold the anode sample, was connected to an analytical balance (Mettler Toledo ML802T, Columbus, OH, SUA), having a precision of 0.01 g, through a steel thread. The total weight of the pannier and the steel thread was 56.36 g. Thermocouples were placed in the steel tube, the oven, and near the anode sample to control the oven temperature, and to measure the oven and sample temperatures. At the top of the tube, a pipe was installed to ventilate the exhausted gas. In order to avoid the oxidation of the sample during the heating period, the bottom of the tube was connected to a support that allows for the supply of the pyrolysis chamber with nitrogen (N_2_) at a flow rate of 2 L/min.

The initial weight of the anode sample was 150.82 g. During the heating period (Figure 2), the released volatile was mixed up with the nitrogen and passed into a glass collector, containing toluene and then placed in a cold water pool, to dissolve condensable gases in the toluene. To fully eliminate the condensable volatile, it was further condensed in a cold water environment and conducted through the drierite (CaSO_4_). Finally, the non-condensable volatile with the nitrogen was ventilated by a vacuum pump at a flow rate of 1.5 L/min. The data were measured at a frequency of 0.2 Hz and then integrated using a LabView software and an acquisition center (DataTaker DT85).

### 2.3. Dilatometry

#### 2.3.1. Device Description and Experimental Procedure

**Figure 4:**
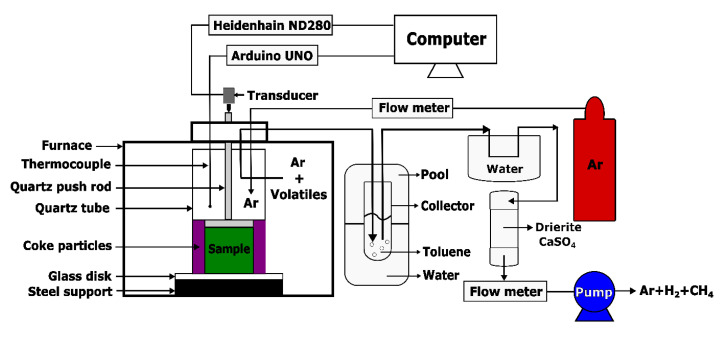
Schematic representation of dilatometry set-up.

The dilatometry test aims to characterize the displacement of the anode in the axial direction at high temperatures for calculating the ratio of apparent volume in Section 3.2.2. The dilatometry was performed using an experimental set-up, which was established in the Aluminium Research Centre—REGAL at Laval University, according to the International Standard ISO 14428 [30], as shown in Figure 4. The set-up consists of a baking furnace, ATS Series 3150 Front Load Box Furnace (ATS, Butler, PA, USA), that can bake the sample up to different target temperatures under 1200 °C. During the baking of the sample in the quartz tube, a light-weight quartz push-rod was used to transmit the vertical displacement of the sample to the transducer that was connected to a Heidenhain (ND280). A thermocouple, which was placed near the sample to measure the sample temperature, was connected to an Arduino UNO serving as a microcontroller board to record the data. A pipe was installed to ventilate the exhausted gas at the top of the furnace. To avoid the oxidation of the sample during the heating period, the sample was surrounded by coke particles (−30 + 50 US mesh) and the argon was injected at a flow rate of 2 L/min.

During the heating period (Figure 2), the released volatile was mixed up with the argon and then passed into a condensation system which used cold water, toluene and drierite (CaSO_4_), as described in Section 2.2. Finally, the non-condensable volatile with the argon were ventilated by a vacuum pump at a flow rate of 1.5 L/min. The data were measured at a frequency of 0.2 Hz and integrated using a Matlab programming system (Appdesigner).

#### 2.3.2. Characterization Method

The vertical strain of the sample was obtained by characterizing the vertical displacement of the sample. However, the components in the apparatus, such as the quartz push-rod, the glass disk, and the steel support, suffer from a thermal expansion that superimposes on the sample displacement. To distinguish the displacement of the sample, the total displacement measured by the transducer should be corrected by extracting the displacement that is caused by the thermal expansion of components in the apparatus, as follows:

(1) $$∆h_{t}^{tot}=∆h_{t}^{D}-∆h_{t}^{S}$$

where $∆h_{t}^{tot}$ is the total vertical displacement of the sample at time t; $∆h_{t}^{D}$ is the total displacement measured by the transducer; and, $∆h_{t}^{S}$ is the displacement that is caused by the thermal expansion of the components in the apparatus.

To characterize $∆h_{t}^{S}$, the equipment was calibrated using a fused quartz glass as the reference material with the same dimensions as the anode sample and a thermal expansion coefficient equals $\alpha_{q}=5.5\;\times 10^{-7}\;{}^{\circ}C^{-1}$. It is calculated, as follows:

(2) $$∆h_{t}^{s}=∆h_{t}^{ct}-∆h_{t}^{q}$$

where $∆h_{t}^{ct}$ is the calibrated displacement that is measured by the transducer and $∆h_{t}^{q}$ is the thermal expansion displacement of the fused quartz glass, which is calculated as:

(3) $$∆h_{t}^{q}=\alpha_{q}∆T\;h_{0}^{q}$$

where ∆T is the temperature difference that is referred to the room temperature, and $h_{0}^{q}=50\;\mathrm{mm}$ is the initial height of the fused quartz glass.

In this way, the total vertical strain of the sample can be obtained by:

(4) $$\varepsilon_{zz}^{tot}=\frac{∆h_{t}^{tot}}{h_{0}^{tot}}$$

where $h_{0}^{tot}$ is the initial height of the sample.

### 2.4. Helium-Pycnometric Measurement of Real Density

The real pynometric density, for different baking temperatures, was measured by a Helium-Pycnometer (Micromeritics, AccuPyc II 1340 (Micromeritics, Norcross, GA, USA) according to the standard ASTM D2638-10 [31]. Note that measurements were made at room temperature. Each test requires 30 g of anode sample powder. For this purpose, a high-energy ball mill (SPEX SamplePrep 8000D Mixer/Miller, Metuchen, NJ, USA) was used to finely grind crushed baked anode samples for 20 min. The powder was dried in an oven (GCA Precision, Gravity Convection Oven Model 16EG (GCA Precision Scientific, Chicago, IL, USA) at 98 °C for a minimum of 8 h to fully eliminate the moisture. Afterwards, it was placed in a desiccator for a minimum of 12 h.

### 2.5. Air Permeability Measurement

The air permeability of a baked anode is measured by determining the resistance to the airflow through a sample slab at room temperature, according to the International Standard ISO 15906 [32]. In the present work, the air permeability of baked-up samples to different high temperatures were measured at the laboratory facilities of Alcoa Deschambault using a RDC-145 (R&D Carbon, Granges, Veveyse, Switzerland). It has a unit of nanoperms (1 nPm = 0.1013 Darcy = 1 × 10^−13^ m^2^).

## 3. Mathematical Models

### 3.1. Baking Index and Shrinking Index

In [13], two variables, called the “baking index” and the “shrinking index”, were defined to characterize the baking level of the anode. The baking index  X characterizes the mass fraction of volatile released in the open pores, while the shrinking index χ characterizes the mass fraction of volatile that is released in the pore space, including the open pores and the closed pores related to the pitch binder. They are defined, as follows:

(5) $$\{\begin{array}{c} X=\frac{m_{vf}}{m_{vf,\infty }}\\ \chi =\frac{m_{vt}}{m_{vt,\infty }} \end{array}$$

where $m_{vf}$ and $m_{vt}$, respectively, represent the mass of volatile that is released in the open pores and pore space, including the open pores and the closed pores related to the pitch binder. The notation “∞” in the subscript denotes the value at the end of the devolatilization process.

The shrinking index deviates from the baking index because of the closed pores related to the pitch binder that entrap the volatile in the carbon anode matrix. It reflects the baking level of the anode mixture in reality. They are linked by a correlation factor ζ, as follows [13]:

(6) $$\{\begin{array}{c} \chi =\frac{X}{\zeta }\\ \zeta =\frac{1+S_{r,\infty }}{1+S_{r}} \end{array}$$

with $S_{r}$ being the ratio of saturation degree, defined as:

(7) $$S_{r}=\tau \frac{\phi_{c}}{\phi_{f}}$$

where τ is the ratio of the volume fraction of the volatile over the corresponding porosities (τ = 1 when the volatile fully saturates the open pores and the closed pores that are related to the pitch binder); $\phi_{c}=\;\phi_{c,i}+\phi_{c,p}$ is the sum of the intergranular and the interfacial porosities ($\phi_{c,i}$) and the bubble closed porosity related to the pitch binder ($\phi_{c,p}$), and $\phi_{f}$ is the open porosity [5]. The ratio of saturation degree $S_{r}$ represents the comparative ratio of closed pores that are related to the pitch binder entrapping the volatile over the open pores transporting the volatile and it reflects the structural transformation in the anode.

### 3.2. Physical Properties

The baking index X can be obtained by the mass loss of the sample. However, several physical properties must be experimentally characterized to obtain the shrinking index  χ.

#### 3.2.1. Real Density

The real density of the anode paste $\rho^{s}$ is defined as:

(8) $$\rho^{s}=\frac{m^{T}}{V^{T}}$$

where $m^{T}$ and $V^{T}$ represent the mass and real volume (without pores) of the paste mixture. Knowing that the anode is composed of the coal tar pitch and filler coke aggregates, the real volume $V^{T}$ is given by:

(9) $$V^{T}=V^{p}+V^{c}=\frac{m^{p}}{\rho^{p}}+\frac{m^{c}}{\rho^{c}}$$

where ${\left(V^{\alpha }\right)}_{\alpha =p,c}$, ${\left(m^{\alpha }\right)}_{\alpha =p,c}$, and ${\left(\rho^{\alpha }\right)}_{\alpha =p,c}$ represent, respectively, the real volume, the mass, and the real density of the phase α (p = pitch, c = coke aggregates).

Let us consider mass ratios of pitch $x^{p}=m^{p}/m^{T}$ and coke aggregates $x^{c}=m^{c}/m^{T}$. They satisfy the relationship $x^{p}+x^{c}=1$. By using Equation (9), one can show that the real density is rewritten as:

(10) $$\rho^{s}=\frac{1}{\frac{x^{p}}{\rho^{p}}+\frac{x^{c}}{\rho^{c}}}$$

Thus, the real density of the anode can be determined by knowing $\rho^{p}$, $\rho^{c}$, and $x^{p}.$

In [33], the real density of the coal tar pitch within the temperature range of 25 °C to 400 °C was found to evolve linearly as a function of the temperature, such that:

(11) $$\rho^{p}=-0.56T+1338.5\;\left(25\;{}^{\circ}C\leq T\leq 400\;{}^{\circ}C\right)$$

The coal tar pitch used in this work is assumed close to the one used in [33].

On the other hand, using the relation $m^{c}=m_{o}^{c}=\rho_{o}^{c}V_{o}^{c}$, the real density of coke aggregates is expressed as:

(12) $$\rho^{c}=\frac{m^{c}}{V^{c}}=\frac{m_{o}^{c}}{V_{o}^{c}+\Delta V^{c}}=\frac{\rho_{o}^{c}}{1+\frac{\Delta V^{c}}{V_{o}^{c}}}$$

where $\rho_{o}^{c}=2.057\;g/{\mathrm{cm}}^{3}$ is the real density of coke aggregates at room temperature [34]; $V_{o}^{c}$ is the initial real volume of coke aggregates; and, $\Delta V^{c}$ is the change of the real volume of coke aggregates due to the thermal expansion. The lowercase “o” denotes the initial value at room temperature.

While using the volumetric thermal expansion relation [35], one can have:

(13) $$\frac{\Delta V^{c}}{V_{o}^{c}}=\exp\left[\int_{T_{o}}^{T}\alpha_{V}^{c}\left(T\right)dT\right]-1$$

where $\alpha_{V}^{c}\left(T\right)$ is the thermal expansion coefficient of coke aggregates. Its value was given in [36]. Thus, the real density of coke aggregates is re-expressed by substituting the Equation (13) into the Equation (12), such that:

(14) $$\rho^{c}=\rho_{o}^{c}\exp\left(-\int_{T_{o}}^{T}\alpha_{V}^{c}\left(T\right)dT\right)$$

The mass ratio of the pitch and mass loss fraction of the anode mixture are rewritten as:

(15) $$\{\begin{array}{l} x^{p}=\frac{m^{p}}{m^{p}+m^{c}}=1-\frac{1}{1+\frac{m^{p}}{m^{c}}}\\ x=1-\frac{m^{T}}{m_{o}^{T}}=1-\frac{1+\frac{m^{p}}{m^{c}}}{1+\frac{m_{o}^{p}}{m_{o}^{c}}} \end{array}$$

Combining these two expressions leads to the following expression of the mass ratio of the pitch:

(16) $$x^{p}=1-\frac{1}{\left(1+\frac{m_{o}^{p}}{m_{o}^{c}}\right)\left(1-x\right)}$$

Therefore, the real density of the anode mixture can be evaluated while using Equations (10), (11), (14), and (16).

#### 3.2.2. Apparent Density

The apparent density of the anode is the mass per unit volume, including all inherent pores within the body of the carbon anode. The apparent density of the anode material strongly influences most of the physical and chemical properties [5]. To estimate their values at high temperatures, the apparent density is first defined as:

(17) $$\rho_{a}^{s}=\frac{m^{T}}{V_{a}^{T}}$$

where $V_{a}^{T}$ is the apparent volume of the anode sample.

By using the Equation (15), the apparent density is rewritten as:

(18) $$\rho_{a}^{s}=\frac{m_{o}^{T}}{V_{a}^{T}}\left(1-x\right)$$

Subsequently, on the right hand side of the Equation (18), if the numerator and the denominator is divided by the initial apparent volume $V_{a,o}^{T}$, the apparent density becomes:

(19) $$\rho_{a}^{s}=\rho_{a,o}^{s}\frac{1-x}{J_{t}}$$

where $\rho_{a,o}^{s}=m_{o}^{T}/V_{a,o}^{T}$ is the initial apparent density and $J_{t}=V_{a}^{T}/V_{a,o}^{T}$ is the ratio of apparent volume expressed as (see Appendix A):

(20) $$J_{t}=1+3\varepsilon_{\mathrm{zz}}^{\mathrm{tot}}$$

#### 3.2.3. Permeability

At high temperatures, it is difficult to measure the anode permeability. Thus, the permeability at high temperatures is expected to be approximated by the air permeability that is measured at room temperature. Furthermore, the return to room temperature after baking does not cause further chemical reactions, hence the porous structure of the material at room temperature is expected to be the same as that at the baking temperature. Therefore, it can be assumed that the evolution of the permeability at high temperatures and the air permeability can have the same varying trend. In this case, a permeability correlator $\gamma_{p}$ is introduced, such that the permeability at high temperatures $k_{p}$ is expressed as:

(21) $$k_{p}=\gamma_{p}\;k_{p,\mathrm{air}}$$

where $k_{p,\mathrm{air}}$ is the air permeability.

#### 3.2.4. Porosities

Total porosity

The total porosity in the carbon anode can be estimated by the apparent density and real density of the anode, as follows [5]:

(22) $$\phi_{p}=1-\frac{\rho_{a}^{s}}{\rho^{s}}$$

Open porosity

During the baking process, the open pores that have a diameter superior to 50 μm influence the permeability of the anode [37]. A Kozeny model using a modified hydraulic radius of the anode particles is used to estimate the open porosity of the anode mixture, as follows [24]:

(23) $$\{\begin{array}{l} k_{p}=\frac{\phi_{f}r_{h,a}^{2}}{k_{o}{\hat{\tau }}^{2}}\\ r_{h,a}=\frac{\phi_{f}}{1-\phi_{f}}\frac{d_{p,a}}{6} \end{array}$$

where $\phi_{f}$ is the open porosity; $k_{o}=2.5$ is the pore shape factor; $\hat{\tau }=1.414$ is the tortuosity; $r_{h,a}$ is the hydraulic radius of the paste particle; and, $d_{p,a}$ is the average diameter of the filler particle sphere, which can be approximated as [38]:

(24) $$d_{p,a}=2r_{p,a}=\frac{2}{N^{t}}\sum_{i=1}^{17}r_{p,a}^{i}N^{i}=198.1\;\mu m$$

where $r_{p,a}$ is the average radius of the filler particle sphere; $N^{t}$ is the total number of the filler particle spheres; $r_{p,a}^{i}$ is the radius of the i-th filler particle sphere; and, $N^{i}$ is the number of the i-th filler particle sphere. In this work, the same recipe of coke particles as that in [38] is used.

The open porosity in the anode can be obtained by the Kozeny model (Equation (23)). To this end, a third-order polynomial equation with respect to the open porosity can be derived:

(25) $$\phi_{f}^{3}-k_{d}{\left(1-\phi_{f}\right)}^{2}=0$$

with

(26) $$k_{d}=\frac{36k_{o}k_{p}{\hat{\tau }}^{2}}{d_{p,a}^{2}}$$

By solving the polynomial Equation (25), the open porosity in the anode can be derived, as follows:

(27) $$\begin{array}{l} \phi_{f}=\frac{1}{3}k_{d}+\frac{1}{6}{\left(-72k_{d}^{2}+108k_{d}+8k_{d}^{3}+12\sqrt{-12k_{d}^{3}+81k_{d}^{2}}\right)}^{1/3}\\ \;\;-\frac{4k_{d}-\frac{2}{3}k_{d}^{2}}{{\left(-72k_{d}^{2}+108k_{d}+8k_{d}^{3}+12\sqrt{-12k_{d}^{3}+81k_{d}^{2}}\right)}^{1/3}} \end{array}$$

Closed porosity

The total closed porosity in the anode $\phi_{c,t}$ can be estimated by extracting the open porosity $\phi_{f}$ from the total porosity $\phi_{p}$, as follows:

(28) $$\phi_{c,t}=\phi_{p}-\phi_{f}$$

Subsequently, the sum of the intergranular, interfacial, and bubble closed porosities that are related to the pitch binder $\phi_{c}$ can be estimated by extracting the closed porosity in filler coke aggregates $\phi_{c,f}$ from the total closed porosity in the anode, as follows:

(29) $$\phi_{c}=\frac{V_{c}}{V_{a}^{T}}=\phi_{c,t}-\phi_{c,f}=\phi_{p}-\phi_{f}-\phi_{c,f}>0$$

where $V_{c}$ is the closed pore volume corresponding to the closed porosity $\phi_{c}$.

Remark: the permeability correlator $\gamma_{p}$ has a maximum value according to the Equation (29) (see Appendix B).

The closed porosity in filler coke aggregates is defined as the ratio of volume that is occupied by the closed pores in filler coke aggregates ($V_{c,f}$) over the apparent volume of the anode:

(30) $$\phi_{c,f}=\frac{V_{c,f}}{V_{a}^{T}}$$

Because the pores in filler coke aggregates within the size range (−50 + 100, −100 + 200, −200 + 400, −400) in US mesh are too small, they can be neglected [34]. The pores in filler coke aggregates within the size range (−4 + 8, −8 + 16, −16 + 30, −30 + 50) are tabulated, as in Table 4.

Let $\phi_{c,f}^{i}$ and $\phi_{t,f}^{i}$ denote, respectively, the closed porosity and total porosity in filler coke aggregates within the i-th size range (US mesh). Moreover, the closed porosity remains constant at high temperatures by assuming the coke aggregates as an isotropic material.

The total closed pore volume in the filler coke aggregates is the sum of each fraction’s individual ones within all size ranges $V_{c,f}=\sum_{i=1}^{4}\phi_{c,f}^{i}V^{i}$, where $V^{i}$ is the apparent volume of filler coke aggregates within the i-th size range. Subsequently, the closed porosity in filler coke aggregates becomes:

(31) $$\phi_{c,f}=\frac{1}{V_{a}^{T}}\sum_{i=1}^{4}\phi_{c,f}^{i}V^{i}$$

The apparent volume of the filler coke aggregates within the i-th size range can be expressed as:

(32) $$V^{i}=\frac{m^{i}}{\rho_{a}^{i}}=m^{T}\frac{\omega^{i}}{\rho_{a}^{i}}$$

where $m^{i}$, $\rho_{a}^{i}$, and $\omega^{i}$ are, respectively, the mass, the apparent density, and the mass fraction of filler coke aggregates within the i-th size range in US mesh. Subsequently, the closed porosity in filler coke aggregates can be rewritten as:

(33) $$\phi_{c,f}=\frac{m^{T}}{V_{a}^{T}}\sum_{i=1}^{4}\phi_{c,f}^{i}\frac{\omega^{i}}{\rho_{a}^{i}}$$

By assuming that the filler coke aggregates within all the size ranges have the same real density $\rho^{c}$, the apparent density of the filler coke aggregates within the i-th size range is expressed as:

(34) $$\rho_{a}^{i}=\rho^{c}\phi_{f}^{i}=\rho^{c}\left(1-\phi_{t,f}^{i}\right)$$

where $\phi_{f}^{i}$ is the real volume fraction of the filler coke aggregates within the i-th size range and $\phi_{t,f}^{i}$ is the total porosity in filler coke aggregates within the i-th size range (US mesh). By recalling the apparent density of the anode $\rho_{a}^{s}=m^{T}/V_{a}^{T}$, the closed porosity in filler coke aggregates can be finally expressed as:

(35) $$\phi_{c,f}=\frac{\rho_{a}^{s}}{\rho^{c}}\sum_{i=1}^{4}\omega^{i}\frac{\phi_{c,f}^{i}}{1-\phi_{t,f}^{i}}$$

#### 3.2.5. Gas Pressure in Closed Pores

The entrapped volatile causes the gas pressure in closed pores. It can be estimated by the baking index and shrinking index. By using Equation (5), the mass of volatile entrapped in the closed pores can be derived:

(36) $$m_{\mathrm{vc}}=m_{\mathrm{vt}}-m_{\mathrm{vf}}=m_{\mathrm{vt},\infty }\left(\gamma_{f,\infty }X-\chi \right)$$

with

(37) $$\{\begin{array}{l} m_{\mathrm{vf}}=m_{o}^{T}-m^{T}\\ m_{\mathrm{vt},\infty }=\alpha_{p,\infty }m_{o}^{p} \end{array}$$

where $\gamma_{f,\infty }=m_{\mathrm{vf},\infty }/m_{\mathrm{vt},\infty }$ is a constant ratio that denotes the mass of volatile released in the open pores at the end of the devolatilization process over the mass of volatile released in the pore space, including the open pores and the closed pores that are related to the pitch binder at the end of the devolatilization process; $\alpha_{p,\infty }$ is the conversion ratio of the pitch at the end of the devolatilization process and $m_{o}^{p}$ is the initial mass of the pitch.

On the other hand, the mass of volatile that is entrapped in the closed pores can be expressed by:

(38) $$m_{\mathrm{vc}}=\rho_{c}^{v}V_{c}$$

where $\rho_{c}^{v}$ denotes the real density of volatile in closed pores corresponding to $\phi_{c}$.

Subsequently, using Equations (29), (36), and (38), and while assuming the volatile follows the ideal gas law $p_{c}=\rho_{c}^{v}\bar{R}T$, the gas pressure in closed pores can be derived:

(39) $$p_{c}=\frac{\bar{R}T}{\phi_{c}V_{a}^{T}}m_{\mathrm{vt},\infty }\left(\gamma_{f,\infty }X-\chi \right)$$

where $\bar{R}=R/M_{v}$ is the specific gas constant that varies with the baking temperature and $M_{v}$ is the molecular weight of the volatile mixture that can be obtained, as follows [23]:

(40) $$M_{v}={\left(\sum_{i=14}\frac{\rho^{v,i}}{\rho^{v}M_{v,i}}\right)}^{-1}={\left(\sum_{i=14}\frac{P_{v,i}}{M_{v,i}}\right)}^{-1}$$

where $\rho^{v,i}$ is the real density of each component of the polycyclic aromatic hydrocarbons (PAHs); $\rho^{v}$ is the real density of gaseous volatile that is approximated by the real density of PAHs; $M_{v,i}$ is the molecular weight of each PAH component; and, $P_{v,i}$ is the concentration of each PAH component.

## 4. Results and Discussion

### 4.1. Thermogravimetric Analysis (TGA)

**Figure 5:**
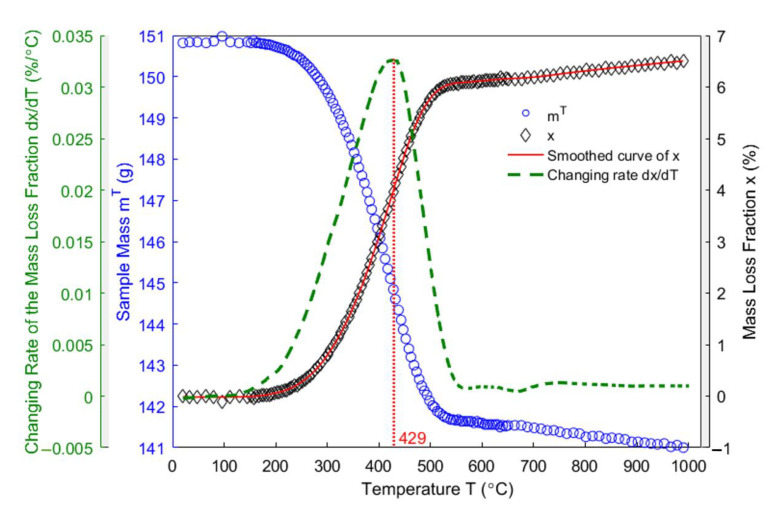
Evolution of the mass, the mass loss fraction and the changing rate of the mass loss fraction of the anode sample.

Figure 5 shows the evolution of the anode mass as a function of the temperature obtained by the thermogravimetric analysis (blue circles) from room temperature to 1000 °C, the corresponding evolution of the mass loss fraction of the anode (black diamonds), and the changing rate of the mass loss fraction (green dashed line). The experimental data are smoothed by using the built-in function ‘rloess’ in Matlab R2016a in order to reduce the oscillation in the mass loss fraction of the anode. Subsequently, the changing rate of the mass loss fraction is obtained as the first derivative of the smoothed curve (red solid line). Observed from the mass loss fraction of the anode, the anode starts losing the weight from around 175 °C, with the mass loss increasing by the rise in the baking temperature. The majority of the volatile is devolatilized from 175 °C to 627 °C, with 6.14% of the sample mass having been evaporated. The maximum rate of the mass loss occurs at 429 °C. The anode’s mass loss slows down during the subsequent baking. At the end of the baking process, the anode loses 9.8 g of mass in form of the volatile, which occupies 6.5% of the total sample mass.

From 527 °C to 627 °C, only 0.11% of mass is lost, which indicates that the pitch carbonization has nearly terminated, and a semi-coke has been formed [39]. Upon further baking from 627 °C to 1000 °C, only 0.36% of total mass will be lost, which indicated that the solid green coke is produced from the semi-coke obtained at the temperatures below 627 °C [39]. According to [20], the volatile emanated by the pitch binder within the temperature range of 175 °C to 527 °C is mainly composed of the condensable gases (PAHs), whereas the methane and hydrogen are mainly degassed from the anode mixture from 527 °C to 1000 °C.

### 4.2. Real Densities

**Figure 6:**
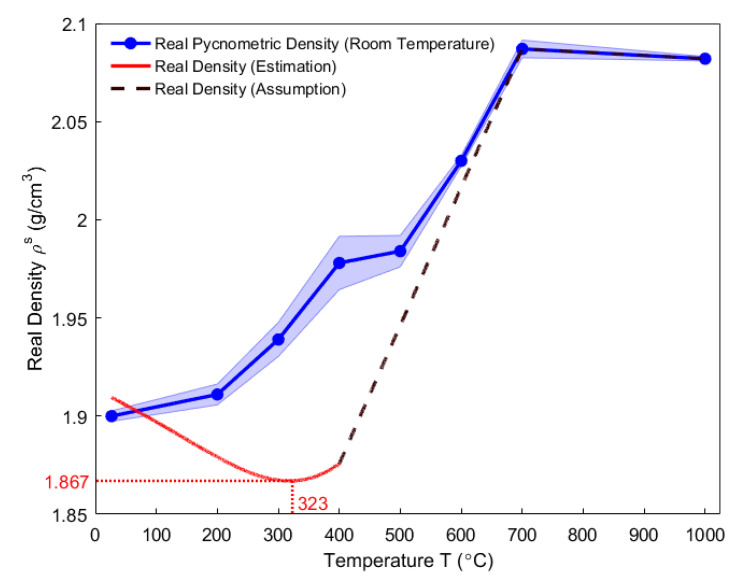
Real pycnometric density measured at room temperature and real density at high temperatures.

In this work, real densities involve the real pycnometric density measured at room temperature and the real density at high temperatures. Real pycnometric density is defined as the ratio of the material mass over the volume that is measured by the helium pycnometer [26]. In Figure 6, the average real pycnometric density of baked-up anode samples to each high temperature (blue solid dots) is calculated by three repeated measurements at room temperature and linearly interpolated. The standard deviations create a shaded error area in which the real pycnometric density likely varies. From room temperature to 700 °C, the real pycnometric density increases from 1.910 g/cm^3^ to 2.082 g/cm^3^. From 700 °C to 1000 °C, the real pycnometric density gains stability, which indicates that the real pycnometric density is neither affected by the demethylation and dehydrogenization processes nor by the growth and reorientation of crystallites [40,41].

The red solid line and the brown dashed line shown in Figure 6 represent, respectively, the real density of the anode from room temperature to 400 °C and from 400 °C to 1000 °C. Below 400 °C, the real density was estimated using Equations (10), (11), (14), and (16). It reached a minimum value of 1.867 g/cm^3^ at 323 °C. Little volatiles devolatilized above 700 °C, hence one can assume that the real density is equal to the real pycnometric density, according to Figure 5. Furthermore, due to the lack of experimental data about the real density of the pitch between 400 °C and 700 °C, it was assumed that the real density would evolve linearly between these two temperatures [3].

Below 175 °C, the thermal expansion and the phase transformation of the pitch take place [40]. This decreases the real density. Between 175 °C and 323 °C, the further decrease in the real density is mainly due to the mass loss from the pitch binder that is caused by the pyrolysis reaction. From 323 °C to 700 °C, the increase in the real density is mainly due to the growth of an intermediate liquid-crystalline structure by consuming the disorganized carbon phase in the pitch binder [3,42].

Nevertheless, one can observe from Figure 6 that there exists a significant difference between the evolutions of the real pycnometric density and the real density of the anode. Moreover, the real pycnometric density is measured at room temperature, where the accuracy of the measurement highly depends on the conditions of preparing the sample powder. Therefore, the real pycnometric density may not reflect the real density of the anode at high temperatures. For this reason, the real density will be used in this work.

### 4.3. Air Permeability

**Figure 7:**
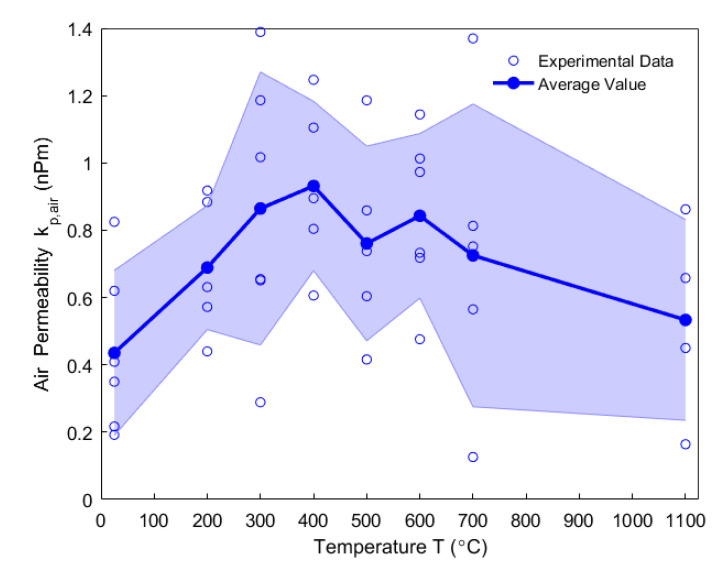
Air permeability of baked-up anodes to different high temperatures.

In Figure 7, the data points of the air permeability are presented by the blue circle dots at different temperatures from 25 °C to 1100 °C. The blue solid dots represent the average air permeability at each temperature. The shaded area corresponds to the standard deviations that vary significantly. Such variations in the standard deviation may be explained by the heterogeneity of porosity and crack distribution within the microstructure of the anode [43]. For this reason, Appendix C presents an analysis of variance (ANOVA) for the data of air permeability. The results of the ANOVA analysis showed that the calculated test statistic F is not far from the critical value of F-distribution at the level of significance 10%. Moreover, such a significant data scattering was also observed in [24] regarding the porosity and the permeability of the anode measured at room temperature, for which the anode samples were baked up to different high temperatures ranging from 300 °C to 1250 °C. In addition, complex thermo-chemo-mechanical transformations would have an impact on the anode during the baking process, such that the anode would not have the same internal structures at high temperatures as those at the beginning of the baking. Therefore, in this work it is assumed that the air permeability should physically evolve with the temperature rather than stay constant.

At room temperature, the air permeability of the green anode is 0.436 nPm. As the temperature rises, it increases by 113.5% up to the maximum 0.931 nPm at 400 °C, where the anode becomes more permeable for the volatile matter to transport and diffuse out of the body volume. The fluctuation of the air permeability from 400 °C to 600 °C might be due to the effects of the thermal expansion, the chemical shrinkage, and the pore pressure of the anode. Form 600 °C to 1100 °C, the air permeability gradually decreases down to 0.534 nPm. It is noteworthy that the prebaked anode has an air permeability that is 22.5% higher than that of the green anode. Because the air permeability is correlated with other measurements, its evolution will be explained in Section 4.4.

### 4.4. Dilatometry

**Figure 8:**
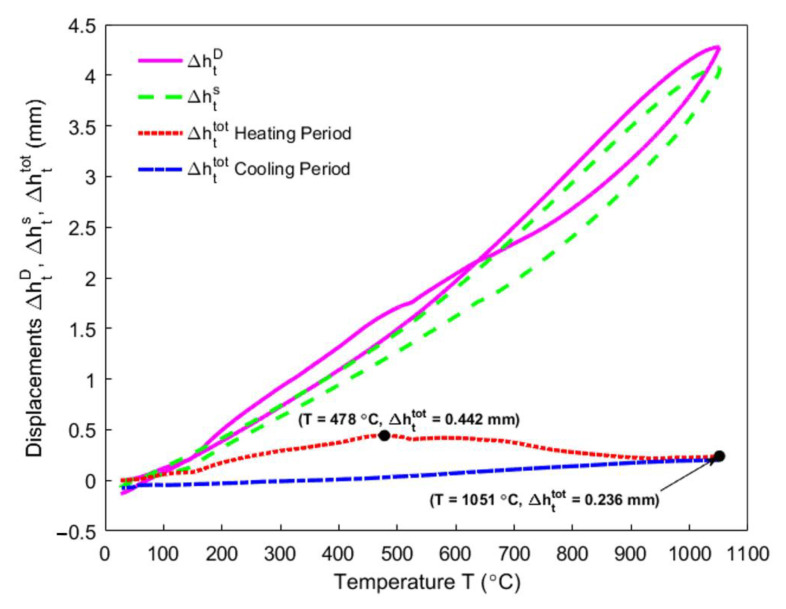
Vertical displacements with respect to the temperature.

The vertical displacements that correspond to $\Delta h_{t}^{D}$, $\Delta h_{t}^{s}$, and $\Delta h_{t}^{\mathrm{tot}}$ were measured by the dilatometry from 25 °C to 1051 °C, as shown in Figure 8. The total displacement of the sample $\Delta h_{t}^{\mathrm{tot}}$ is obtained by the difference between the total displacement measured by the transducer $\Delta h_{t}^{D}$ (purple solid line) and the displacement that is caused by the thermal expansion of the components in the apparatus $\Delta h_{t}^{s}$ (green dashed line). The red dotted line and the blue dash-dot line, respectively, represent the total displacement of the sample during the heating and the cooling period.

**Figure 9:**
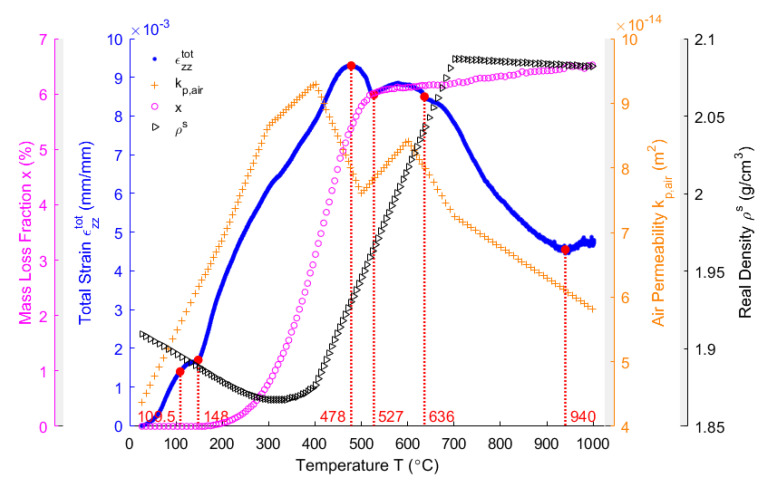
Evolution of the total strain of the anode sample in the vertical direction, the mass loss fraction, the air permeability, and the real density of the anode sample.

It can be observed that the total displacement of the sample during the heating period reaches a maximum value of 0.442 mm at 478 °C. Above 478 °C, it decreases to 0.236 mm at the end of the heating period (1051 °C). By using Equation (4), the total strain of the sample in the vertical direction $\varepsilon_{\mathrm{zz}}^{\mathrm{tot}}$ is obtained, which is shown in Figure 9 (blue points). It is presented accompanied by the other properties, such as the mass loss fraction x (purple circles), the air permeability $k_{p,\mathrm{air}}$ (orange plus signs), and the real density $\rho^{s}$ (black right-pointing triangles).

In Figure 9, as observed from the total strain of the anode sample in the vertical direction, the sample expands due to the thermal expansion of solid pitch and coke aggregates below the pitch softening point of 109.5 °C. When the temperature exceeds 109.5 °C, the pitch melts into a liquid releasing the tensions that formed by the green sample compaction, such that the sample is plasticized [40]. The tension release might cause a body collapse in the plastic anode, which slows down the thermal expansion from 109.5 °C to 148 °C. Between 175 °C (starting of the pitch pyrolysis) and 478 °C, the pitch pyrolyzes (purple circles) and part of the released volatile is entrapped in the closed pore volume of the anode [6]. This process might dominate the sharp increase of the total strain in this temperature range. However, the air permeability (orange plus signs) simultaneously increases until reaching its maximum at 400 °C, which practically coincides with when the maximum changing rate of the mass loss fraction is reached (Figure 5), and indicates that greater open pore volume is produced for the volatile to discharge out at this moment. This necessitates investigating the comparative significance of the closed pore volume over the open pore volume within this temperature range, which will be addressed in subsequent sections. The total strain reaches its maximum at 478 °C, when the air permeability declines from 400–500 °C. This might be due to the increase in the closed pore volume. From 478–527 °C, the slight shrinking of the material is due to the decrease in the changing rate of the mass loss fraction (Figure 5), which indicates that the released volatile lacks enough power to maintain the expansion of the anode sample [3]. Nevertheless, a hump of the total strain of the anode sample takes place between 527–636°C, and it hinders the shrinkage, even though the air permeability increases from 500–600 °C. This might be due to little volatile that is degassed from the open pore volume and the entrapment of the volatile in the closed pore volume. This will be highlighted together with the gas pressure in closed pores shown in Section 4.8. According to [16], the pitch remains in liquid state as far as 527 °C, with the shrinkage after 582 °C in the middle of the hump being due to the pitch carbonization into a semi-coke. Above 627 °C, the solid green coke is formed by the semi-coke due to the pitch carbonization. From 636–940 °C, the chemical shrinkage of the solid green coke causes a pronounced shrinkage of the apparent volume of the material, such that the air permeability decreases. Above 940 °C, the material gains a high mechanical strength [44], owing to the pre-graphitization. This allows for the material to resist the chemical shrinkage. Thus, the small increase in the total strain is due to the thermal expansion.

**Figure 10:**
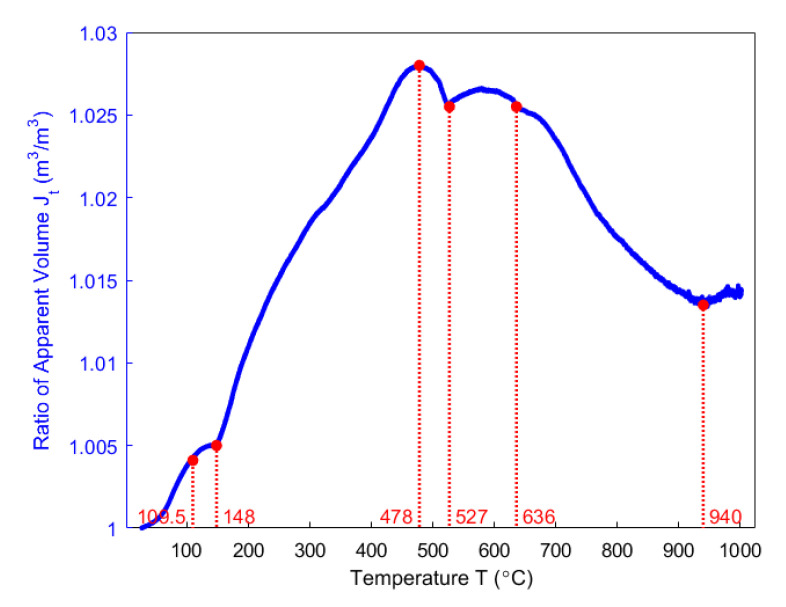
The evolution of the ratio of apparent volume as a function of temperature.

By using Equation (20), the ratio of apparent volume $J_{t}$ during the heating period is obtained, as shown in Figure 10.

### 4.5. Apparent Density

**Figure 11:**
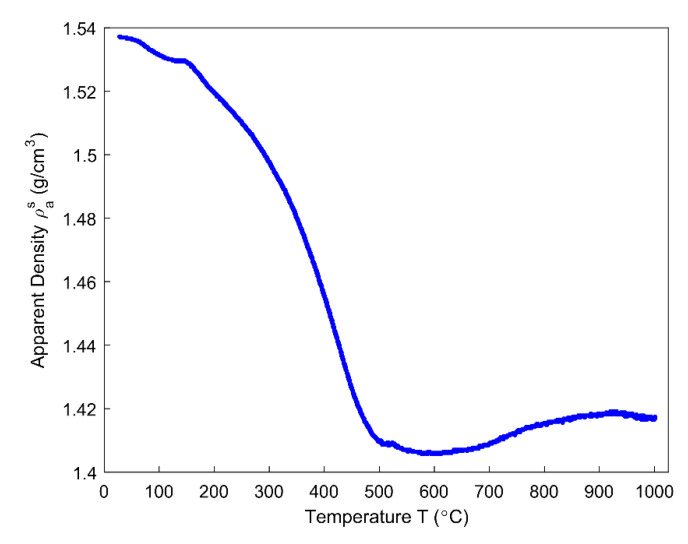
The evolution of apparent density as a function of temperature.

The apparent density is obtained by Equation (19), as presented in Figure 11. It has a minimum value of 1.406 g/cm^3^ at 577 °C. Below 577 °C, the apparent density of the anode rapidly decreases mainly due to the increase in the mass loss (Figure 5) and the ratio of apparent volume (Figure 10). From 577–1000 °C, the apparent density increases since the variation of the mass loss is small and the ratio of apparent volume significantly decreases. It is noticed that the temperature of the minimum apparent density practically lies in the temperature range of 527–636 °C, where the summit of the hump taking place, as shown in Figure 10. Therefore, the apparent density of the anode reaches the minimum neither at which appears the highest changing rate of mass loss fraction at 429 °C (Figure 5) nor appears the highest ratio of apparent volume at 478 °C (Figure 10). However, the minimum apparent density that is reached at 577 °C seems to be due to the pitch carbonization into the semi-coke, as mentioned in [39].

### 4.6. Porosities

**Figure 12:**
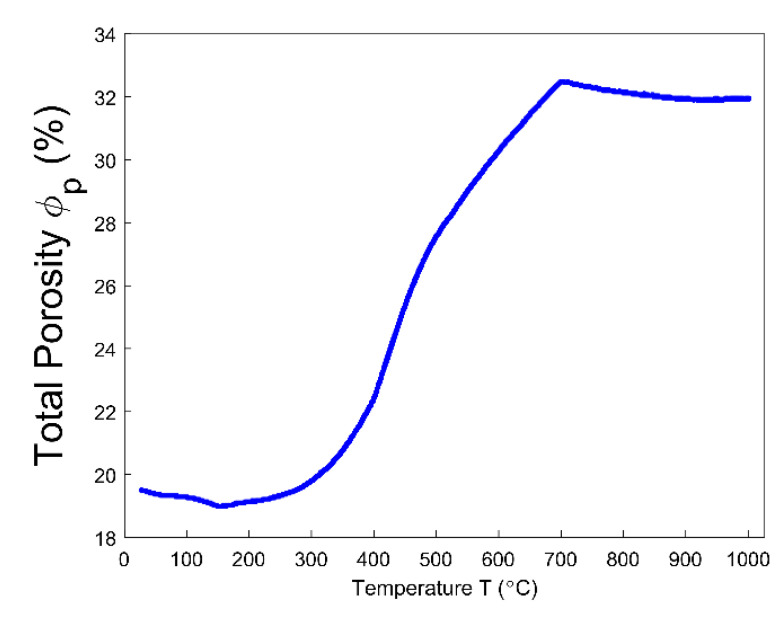
Total porosity.

**Figure 13:**
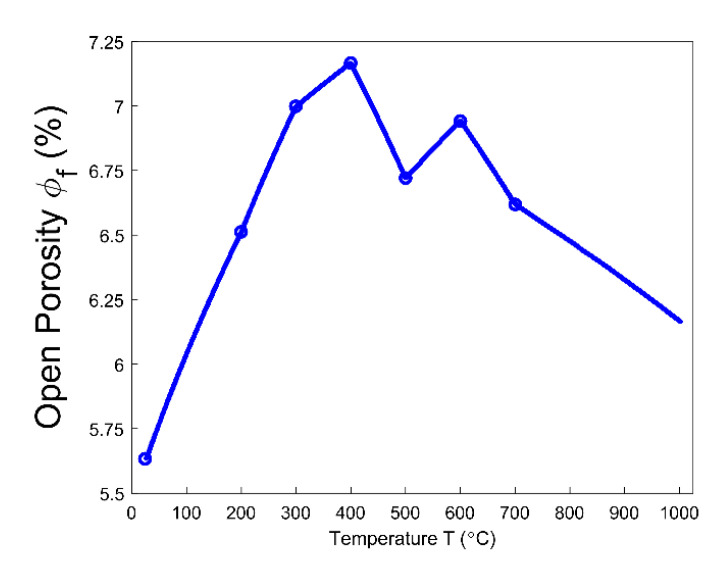
Open porosity.

**Figure 14:**
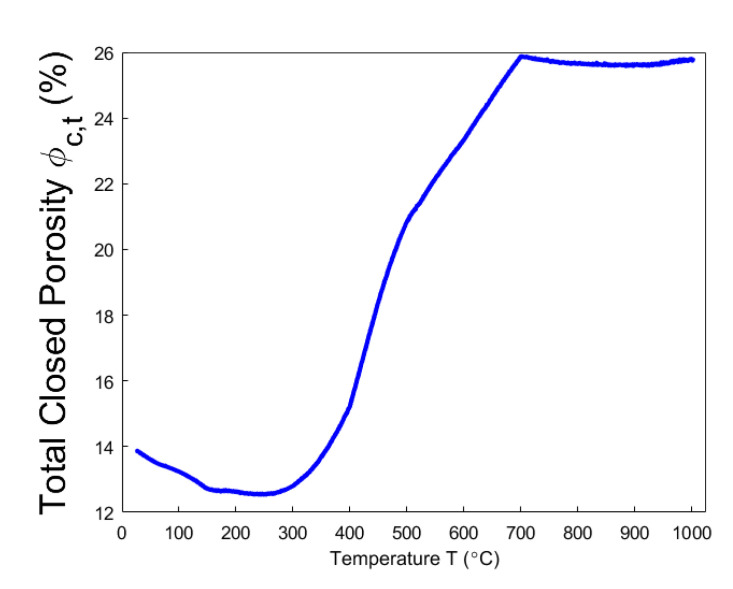
Total closed porosity.

**Figure 15:**
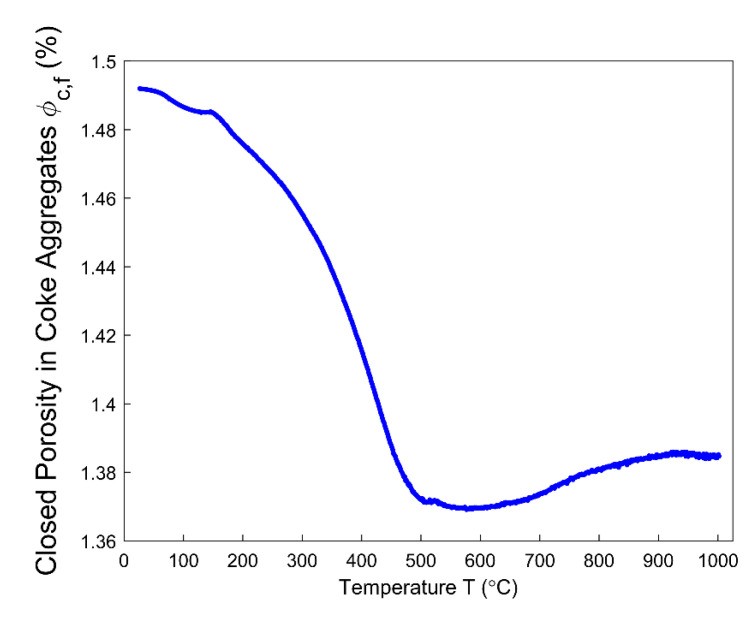
Closed porosity in filler coke aggregates.

**Figure 16:**
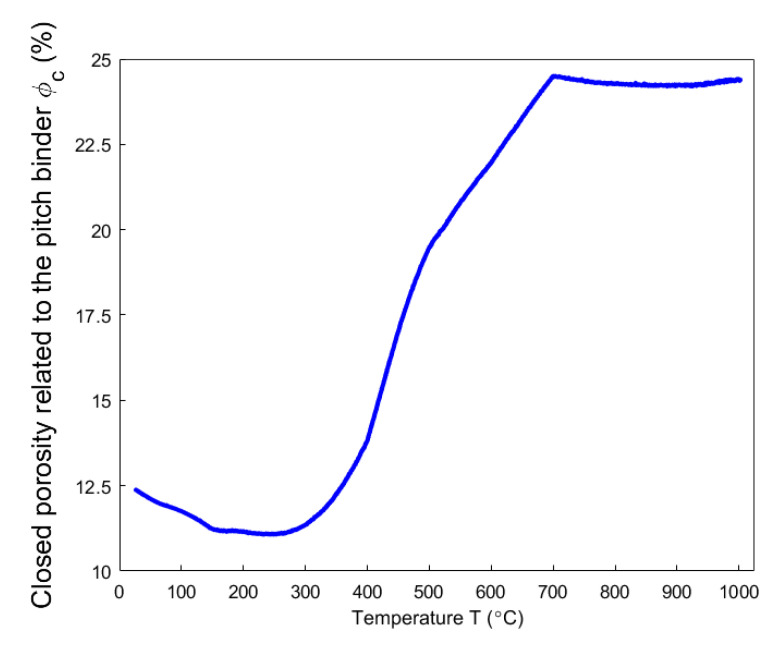
Sum of intergranular, interfacial and bubble closed porosities related to the pitch binder.

Even though the ratio of apparent volume has been determined, the way in which the porous structure transforms in the anode still remains not fully understood. For this reason, different porosities are respectively presented in Figure 12, Figure 13, Figure 14, Figure 15 and Figure 16. First, the total porosity is obtained through the real density and the apparent density, by using the expres Equation (22), as shown in Figure 12. The total porosity of the anode decreases until reaching a minimum value of 19% at 154 °C. From 154–700 °C, it sharply increases by 13.5%. From 700–1000 °C, the total porosity is almost constant. Second, by using the Equation (27), the open porosity is obtained based on the permeability at high temperatures, as shown in Figure 13. To this end, it was assumed that the permeability at high temperatures coincides with the measured air permeability ($\gamma_{p\;}=1.00$ in Equation (21)). The discontinuous points of open porosity are linearly interpolated to be consistent with the continuous results of total porosity presented in Figure 12. It has a maximum value of 7.1% at 400 °C. The increase between 175–400 °C mainly results from the increase in the mass loss of the anode within this temperature range (Figure 5). The following decrease from 400–500 °C might be due to the accumulation of bubble closed pores in the pitch binder, which lack sufficient passages to the open porous network. From 500–600 °C, the chemical shrinkage of the pitch binder links the open pores with the bubble closed pores [9]. Consequently, the volatiles that are entrapped in bubble closed pores are released into the open pores, which facilitates the outflow of the volatiles and increases the open porosity by increasing the gas pressure in the open pores. Above 600 °C, the decrease of the open porosity is again mainly caused by the chemical shrinkage of the semi-coke or solid green coke closing up the open pore volume. Third, the total closed porosity is calculated by the difference between the total porosity and open porosity. It has a minimum value of 12.5% at 208 °C, as presented in Figure 14. In order to estimate the porosity which mainly entraps the volatile during the baking process, the closed porosity in filler coke aggregates is reached by the data in Table 4, Equations (14) and (35), as shown in Figure 15. It is considerably influenced by the apparent density of the anode, since it mainly decreases when the apparent volume of the anode increases. It has a minimum value of 1.37% at 577 °C and its total variation is small under 1.5%. Because the closed pores in filler coke aggregates do not entrap or transport any released volatile during the baking process, they only trivially affect the total closed porosity of the anode. Thus, the change of total closed pores in Figure 14 mainly results from the intergranular, interfacial, and bubble closed pores related to the pitch binder. The sum of their corresponding porosities, as presented in Figure 16, is obtained based on the difference between the total closed porosity (Figure 14) and the closed porosity in filler coke aggregates (Figure 15). It plays an important part in the anode volume change and the obstruction of the volatile from being released out of the material. During the baking process, the open pore volume of the anode (Figure 13) occupies between 5.6% and 7.1% of the anode volume. This is much less than the closed pore volume (Figure 16), which varies between 11% and 24.5% of the anode volume. Such a variation indicates that the volume fraction of the total pore space and the change in porous structures of the anode are mainly affected by the closed pores that are related to the pitch binder. The sum of intergranular, interfacial, and bubble closed porosities related to the pitch binder has a minimum value of 11% at 208 °C, as shown in Figure 16. From 25 °C to 208 °C, by knowing that the amount of released volatile is negligible (Figure 5), the decrease in the amount of closed porosities that are related to the pitch binder is mainly due to the pitch softening in this temperature range, since the flow and expansion of the liquid pitch closes the interfacial pores and seals the open pores of the coke aggregates forming the intergranular pores. Moreover, according to Figure 12, the variation of total porosity is small in this temperature range. The open porosity in this temperature range increases by around 1% (Figure 13), while the sum of closed porosities related to the pitch binder in Figure 16 decreases by around 1.5%. This indicates that there might also exist an opening of the closed pores related to the pitch pyrolysis. From 208–400 °C, the closed porosity that is related to the pitch binder increases by 3%, which is more significant than the increase in the open porosity, i.e., by 0.6%. This implies that, apart from the thermal expansion of the granular carbon matrix [36], the increase in the total strain of the anode sample from 208–400 °C in Figure 9 is mainly caused by the accumulation of globular pores in the pitch binder created by the bubble gaseous volatile. Additionally, it is also affected by the volume expansion of the intergranular and interfacial pores due to the packing and the sealing of the pitch around the coke aggregates prior to its carbonization [6]. From 400–700 °C, the accumulation of globular pores significantly increases, as does the volume of the intergranular and interfacial pores [6]. In this temperature range, this leads to the sharp increase in the sum of the corresponding porosities, as shown in Figure 16. Above 700 °C, because the matrix of the carbon anode is strengthened by the solidification of the pitch binder and the majority of volatile has been discharged out of the pore space, the closed pore volume that is related to the pitch binder remains largely unaffected by the baking process.

**Figure 17:**
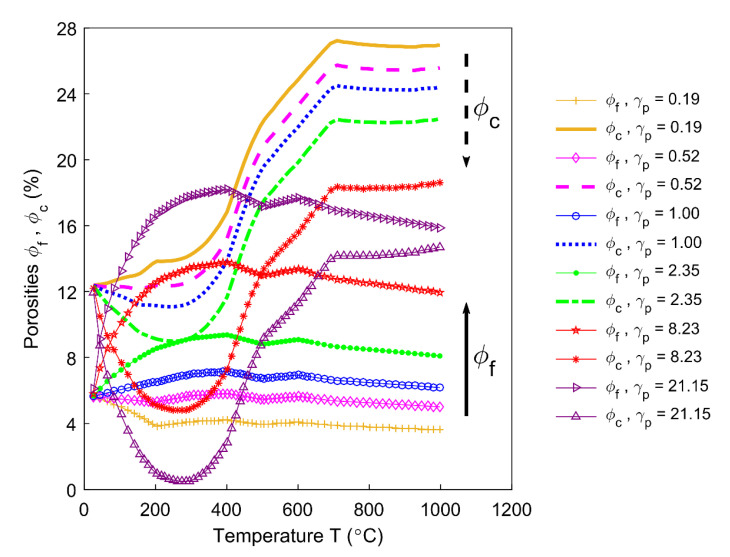
Sensitivity analysis of porous structures for different permeability correlators.

The permeability at high temperatures would quite possibly deviate from the air permeability that is measured at room temperature, such that the accuracy of estimating the open porosity of the anode would be significantly affected. This deviation is evaluated by the permeability correlator $\gamma_{p}$, which will make a difference to the profile of the porous structure (Equation (21)). For this purpose, a sensitivity analysis of the open porosity $\phi_{f}$ and closed porosity $\phi_{c}$ is performed for different permeability correlators, as shown in Figure 17. When the permeability at high temperatures equals the air permeability $\gamma_{p}=1.00$, the open porosity $\phi_{f}$ (blue circle line) and the closed porosity $\phi_{c}$ (blue dotted line) have been displayed in Figure 13; Figure 16. When $\gamma_{p}$ changes from 0.19 to 2.35, the magnitude of $\phi_{c}$ decreases, but it is still higher than $\phi_{f}$, which indicates that the material inclines to entrap the volatile. When $\gamma_{p}$ changes from 2.35 to 21.15, the magnitude of $\phi_{c}$ diminishes and $\phi_{c}$ decreases more rapidly before reaching its minimum value. Moreover, it equals $\phi_{f}$ at certain temperatures and the relative predominance of $\phi_{c}$ over $\phi_{f}$ is considerably weakened. At this moment, the material becomes inclined to release the volatile.

### 4.7. Shrinking Index and Baking Index

**Figure 18:**
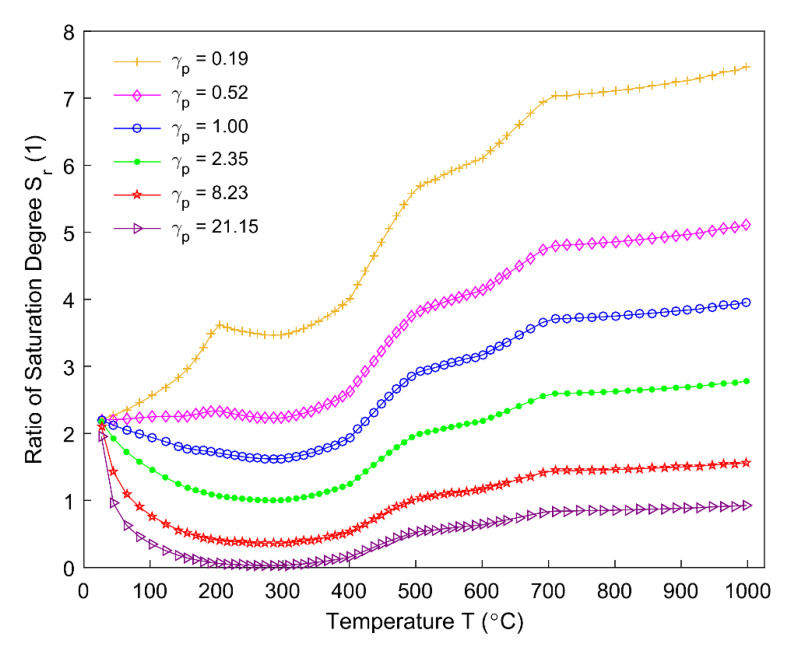
Ratio of saturation degree for different permeability correlators.

**Figure 19:**
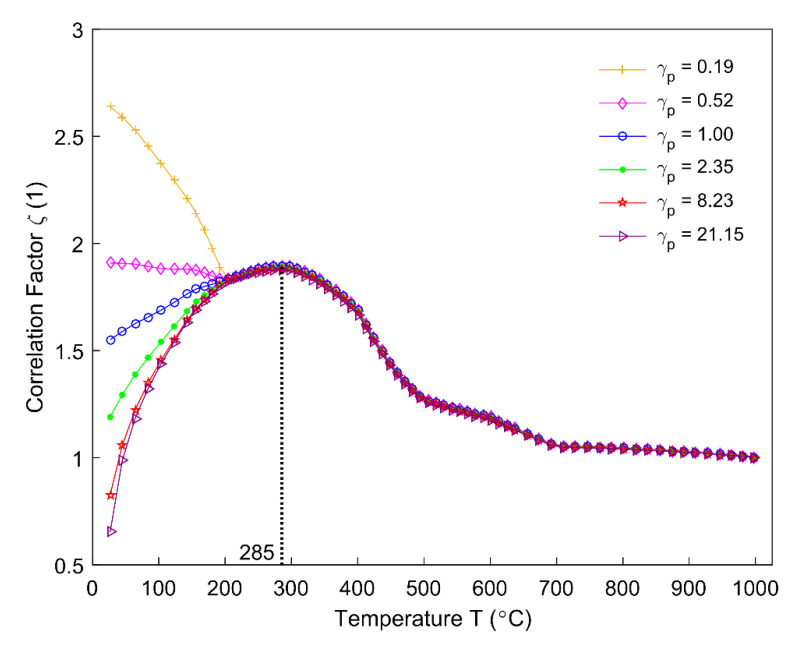
Correlation factor for different permeability correlators.

The ratio of saturation degree $S_{r}$ in the Equation (7) and correlation factor ζ in the Equation (6) for different permeability correlators $\gamma_{p}$ are obtained, as shown, respectively, in Figure 18; Figure 19. The ratio of saturation degree is sensitive to the permeability correlator. With an increase in the permeability correlator, the material system becomes more permeable and allows more degassing of the volatile (Equation (7)), as seen in Figure 18. Figure 19 shows that the correlation factor ζ is sensitive to the permeability correlator below 200 °C, while it becomes insensitive as soon as the temperature rises above 200 °C. During the pyrolysis process, the correlation factor is practically unaffected as to whether the anode material is permeable or obstructive for the gaseous volatile, and it reaches its maximum value around 285 °C in the region where ζ is insensitive to $\gamma_{p}$.

**Figure 20:**
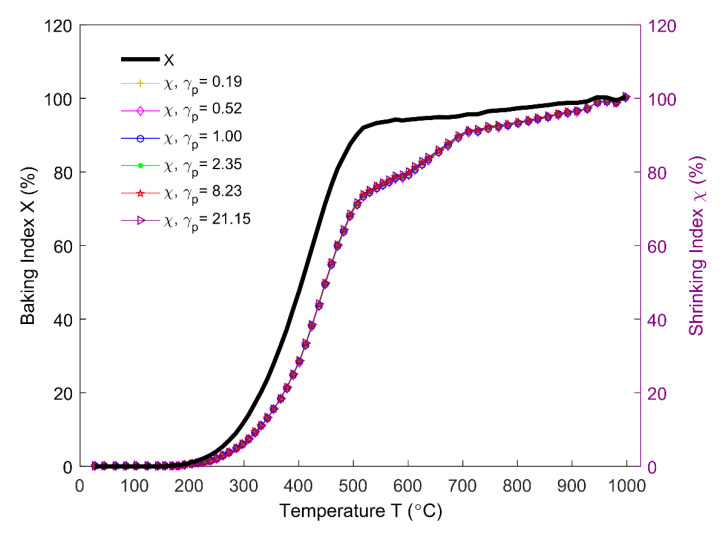
Baking index and shrinking index for different permeability correlators.

The baking index that is displayed in Figure 20 (black solid line) is obtained by using the sample mass (Equation (5)), as shown in Figure 5. The shrinking index is estimated by Equations (6) and (7) for different permeability correlators $\gamma_{p}$. The shrinking index is insensitive to permeability at high temperatures, as can be seen from Figure 20. Even though the correlation factor ζ undergoes a large variation for different permeability correlators below 200 °C, the shrinking index remains stable, since the amount of volatile released below 200 °C is trivial. Between 175 °C (starting of the pitch pyrolysis) and 1000 °C, it is observed that the shrinking index characteristically evolves in the area under the baking index. This phenomenon could be explained by the existence of the closed pores that are related to the pitch binder in the anode. Above 175 °C, the entrapment of the released volatile in the intergranular, interfacial, and bubble closed pores causes a gradual deviation of the shrinking index from the baking index. The maximum difference can reach as much as 22% at 454 °C. Therefore, at a given temperature, it can be seen that the baking index that is observed by the thermogravimetric analysis overestimates the baking level of the anode.

### 4.8. Gas Pressure in Closed Pores

It is vital to investigate the gas pressure in these pores since the volatile trapped in closed pores related to the pitch binder can have a significant impact on the apparent volume of the anode [6]. However, these pores are inaccessible, and they transform with a lot of uncertainty during the baking process. Thus, measuring the gas pressure in these pores by available apparatus entails much difficulty at present temperature. Nonetheless, the gas pressure in these pores can be approximated employing the ideal gas law (Equation (39)).

**Figure 21:**
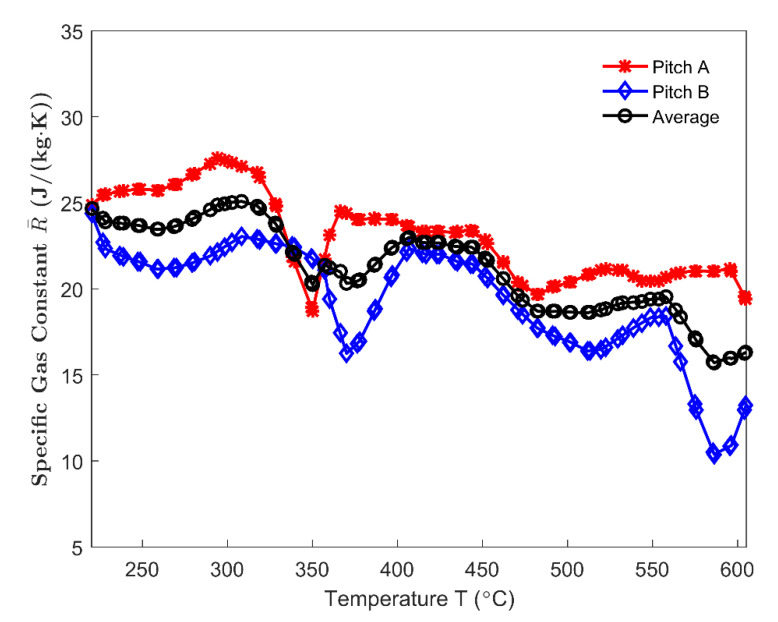
Specific gas constant from 220 °C to 605 °C.

The specific gas constant $\bar{R}$ in the Equation (39) is needed to estimate the gas pressure in closed pores. The specific gas constant of the condensable volatile can be obtained by using the molecular weight of the condensable volatile mixture (Equation (40)). Moreover, it is assumed that the pitch that is used in this work has properties that are similar to those of the pitch used in [27]. In [27], the concentration of each component of polycyclic aromatic hydrocarbons (PAHs) from 220–605 °C, given that their molecular weights are known, were obtained by the chromatographic analysis for two types of pitches A and B. Accordingly, the specific gas constant for pitches A (red asterisk line) and B (blue diamond line) and their average value (black circle line) are obtained and shown in Figure 21.

The pitch quantity converted to the volatile at the end of the baking process corresponds to 46.63%, according to Figure 5 and knowing that the quantity of pitch occupies 13.94% of the initial sample mass. Thus, the conversion ratio of pitch $\alpha_{p,\infty }$ in the Equation (37) equals 46.63%. In [19], the conversion ratio of a pure coal tar pitch approximately equals 46%. This indicates that, at the end of the devolatilization process, the volatile released from the carbon matrix has almost fully escaped from the anode, namely, $m_{\mathrm{vt},\infty }\approx m_{\mathrm{vf},\infty }=9.8\;g$ and $\gamma_{f,\infty }\approx 1$. Thus, the mass of volatile remained in the closed pores that are related to the pitch binder at the end of the devolatilization process can be neglected and the Equation (39) for gas pressure in these pores is rewritten as:

(41) $$p_{c}=\frac{\bar{R}T}{\phi_{c}V_{a}^{T}}m_{\mathrm{vf},\infty }\left(X-\chi \right)$$

**Figure 22:**
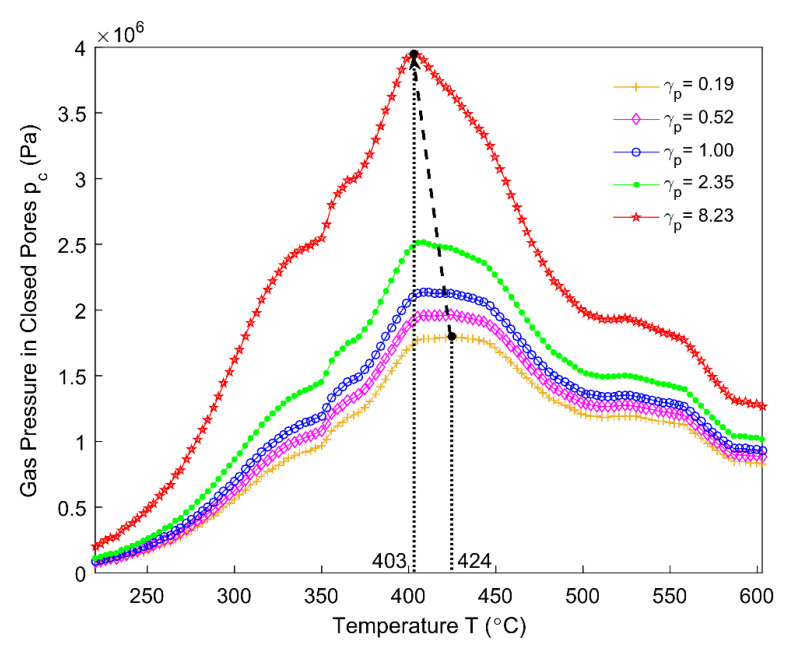
Gas pressure in closed pores for different permeability correlators.

By using the average specific gas constant presented in Figure 21, the gas pressure in closed pores is estimated from 220 °C to 605 °C for different permeability correlators $\gamma_{p}$ in Figure 22. The pore pressure corresponding to the permeability correlator $\gamma_{p}=21.15$ has been neglected, since the corresponding $\phi_{c}$ almost vanishes at 278 °C (Figure 17). With the increase in the permeability correlator, the magnitude of the pore pressure increases and the maximum value moves backwards, which indicates that the gas pressure in closed pores related to the pitch binder is sensitive to the permeability at high temperatures. The maximum pressure appears within the temperature range of 400–450 °C, which nearly coincides with the maximum releasing rate of the mass loss and maximum permeability.

Within the temperature range of 148–478 °C, the ratio of apparent volume increases and reaches the maximum at 478 °C, as shown in Figure 10. It can be seen in Figure 22 that, for $\gamma_{p}=1.00$, the gas pressure in closed pores increases between the temperature range of 220 °C to 424 °C, in which the majority of volatile is entrapped in the closed pore volume. Thus, the gas pressure in closed pores significantly affects the apparent volume of the anode from 220–424 °C. Beyond 424 °C, the gas pressure decreases, which indicates that the closed pores that are related to the pitch binder have been connected with the open pores around 424 °C and the volatile entrapped in closed pores is being released. Furthermore, even though the gas pressure in closed pores decreases between 527–605 °C (Figure 22), it is still high. However, the mass loss from the anode is small within this temperature range (Figure 5). This could explain the hump of the ratio of apparent volume between 527–636 °C seen in Figure 10. On the other hand, the gas pressure in closed pores related to the pitch binder will become nearly zero at the end of the baking process (χ≈X), which implies that the volatile in these pores will be almost fully effused out at the end of the baking process, according to Figure 20 and the Equation (41).

## 5. Conclusions

In this work, some relevant physical properties of the anode during the baking process were characterized. To this end, experiments that consist of thermogravimetric analysis, dilatometry, air permeability, and helium-pycnometric measurements were carried out.

The obtained results allow for the characterization of the mass loss of the anode, the real pycnometric density, the air permeability, and the ratio of the apparent volume. The real density of the anode, at high temperatures, was estimated using real densities of the coal tar pitch and coke aggregates. It was shown that the real and apparent densities of the anode at high temperatures were both significantly impacted by the mass loss of the anode due to the chemical reaction of pitch pyrolysis. Moreover, the real density of the anode at high temperatures differs from the real pycnometric density of the anode measured at room temperature, and the apparent density of the anode was also affected by the apparent volume change of the anode to a large extent.

The permeability at high temperatures was linked to the air permeability through a permeability correlator, due to difficulties that are related to its measurement at high temperatures. Afterwards, the open porosity and the closed porosity that were associated with the pitch binder were estimated. A sensitivity analysis based on the permeability correlator showed that these porosities are linked closely to the permeability at high temperatures.

A new variable to characterize the baking level of the anode, called the shrinking index, was introduced and estimated. It was shown that the shrinking index was not sensitive to the permeability correlator associated with the permeability at high temperatures. Moreover, the closed porosity that is associated with the pitch binder deviates the shrinking index from the baking index, which was observed by the thermogravimetric analysis (TGA). Therefore, the shrinking index reflects the baking level of the anode in reality. Employing the aforementioned results, the gas pressure in closed pores related to the pitch binder was estimated. The results showed that the magnitude of gas pressure in closed pores related to the pitch binder is sensitive to the permeability correlator. Furthermore, the evolution of the gas pressure in closed pores related to the pitch binder indicated that the open pores and closed pores related to the pitch binder would be connected between 400 °C and 450 °C, which led to a decrease in the gas pressure in closed pores related to the pitch binder after reaching its maximum value. While using the ratio of apparent volume, the analysis of these results also provided some insights into the swelling and shrinkage phenomena affecting the anode during the baking process.

These investigations pave the way for studying the chemical effect of the pitch pyrolysis on the mechanical properties of the anode under external loading confinements. Further experimental works are needed to characterize the mechanical behaviors of the anode.

## Figures and Tables

**Table 1:** Size distribution of coke aggregates in anode samples [26].

Size Range (US Mesh)	Mass (wt.%)
−4 + 8	22.0
−8 + 14	10.0
−14 + 30	11.5
−30 + 50	12.7
−50 + 100	8.8
−100 + 200	10.8
>−200	24.2

**Table 2:** Properties of the coal tar pitch used as binder phase in anode samples [26,28].

Softening Point (°C)	Quinoline Insoluble (%)	Coking Value (%)
109.5	16.5	58.8

**Table 3:** Sample dimensions for different characterizations.

Characterizations	Type	Diameter × Height (mm × mm)
Thermogravimetric analysis	Green	50 × 50
Dilatometry		50 × 50
Helium-pycnometric measurement	Baked	-
Air permeability measurement		50 × 20

**Table 4:** Size and mass fraction of filler coke aggregates in anode paste sample [34].

No.	Size Range (US Mesh)	Mass Fraction $\omega^{i}$ (wt.%)	Closed Porosity of Filler Coke Aggregates (%)	Open Porosity of Filler Coke Aggregates (%)
1	−4 + 8	22	2.33594	31.2216
2	−8 + 16	10	2.45957	26.8466
3	−16 + 30	11.5	2.83879	19.9858
4	−30 + 50	12.7	2.87717	16.3068

## Data Availability

Data sharing is not applicable to this article.

## Appendix A. Calculation of the Ratio of Apparent Volume

The sample was cylindrical. In order to estimate the ratio of apparent volume, let us first calculate the variation of the apparent volume as:

(A1) $$\Delta V_{a}^{T}=V_{a}^{T}-V_{a,o}^{T}=\frac{\pi }{4}{\left(d_{o}^{\mathrm{tot}}+\Delta d_{t}^{\mathrm{tot}}\right)}^{2}\left(h_{o}^{\mathrm{tot}}+\Delta h_{t}^{\mathrm{tot}}\right)-\frac{\pi }{4}d_{o}^{\mathrm{tot}2}h_{o}^{\mathrm{tot}}\approx \frac{\pi }{4}d_{o}^{\mathrm{tot}2}\Delta h_{t}^{\mathrm{tot}}+\frac{\pi }{2}d_{o}^{\mathrm{tot}}h_{o}^{\mathrm{tot}}\Delta d_{t}^{\mathrm{tot}}$$

where $d_{o}^{\mathrm{tot}}$ is the initial diameter of the anode sample; $\Delta d_{t}^{\mathrm{tot}}$ is the total displacement of the anode sample in the radial direction and $\Delta h_{t}^{\mathrm{tot}}$ is the total displacement of the sample in the axial direction.

Then, the apparent volume change can be expressed as a function of the vertical strain component and the radial strain component, as follows:

(A2) $$\Delta V_{a}^{T}=\left(\frac{\Delta h_{t}^{\mathrm{tot}}}{h_{o}^{\mathrm{tot}}}+2\frac{\Delta d_{t}^{\mathrm{tot}}}{d_{o}^{\mathrm{tot}}}\right)V_{a,o}^{T}=\left(\varepsilon_{\mathrm{zz}}^{\mathrm{tot}}+2\varepsilon_{\mathrm{rr}}^{\mathrm{tot}}\right)V_{a,o}^{T}$$

where $\varepsilon_{\mathrm{rr}}^{\mathrm{tot}}$ is the total strain in the radial direction.

By assuming the material is isotropic and knowing that the anode sample is free of mechanical loading in dilatometry, the vertical and radial strain component equal as $\varepsilon_{\mathrm{zz}}^{\mathrm{tot}}=\varepsilon_{\mathrm{rr}}^{\mathrm{tot}}$. Subsequently, the apparent volume change can be rewritten as:

(A3) $$\Delta V_{a}^{T}=3\varepsilon_{\mathrm{zz}}^{\mathrm{tot}}V_{a,o}^{T}$$

The ratio of apparent volume can be consequently obtained as:

(A4) $$J_{t}=\frac{V_{a}^{T}}{V_{a,o}^{T}}=\frac{V_{a,o}^{T}+\Delta V_{a}^{T}}{V_{a,o}^{T}}=1+3\varepsilon_{\mathrm{zz}}^{\mathrm{tot}}$$

## Appendix B. Extremity of the Permeability Correlator

This appendix aims to obtain the maximum value of the permeability correlator. By using Equations (23) and (21), the permeability correlator can be rewritten as:

(A5) $$\gamma_{p}=\frac{d_{p,a}^{2}}{36k_{o}{\hat{\tau }}^{2}k_{p,\mathrm{air}}}\frac{\phi_{f}^{3}}{{\left(1-\phi_{f}\right)}^{2}}$$

The first derivative of the permeability correlator with respect to the open porosity can be derived:

(A6) $$\frac{\partial \gamma_{p}}{\partial \phi_{f}}=\frac{d_{p,a}^{2}}{36k_{o}{\hat{\tau }}^{2}k_{p,\mathrm{air}}}\frac{\phi_{f}^{2}\left(3-\phi_{f}\right)\left(1-\phi_{f}\right)}{{\left(1-\phi_{f}\right)}^{4}}>0$$

According to the Equation (29), when the closed porosity $\phi_{c}$ reaches its minimum value at a certain temperature, the open porosity reaches its maximum value as:

(A7) $$\phi_{f}^{\max}={\left(\phi_{p}-\phi_{c,t}\right)|}_{T_{f}^{\max}}$$

where $T_{f}^{\max}$ is the temperature at which the open porosity reaches its maximum value.

From the Equation (A6), the permeability correlator should monotonously increase with respect to the open porosity. Thus, according to the Equation (A5), the maximum permeability correlator can be obtained, as follows:

(A8) $$\gamma_{p}^{\max}=\frac{d_{p,a}^{2}}{36k_{o}{\hat{\tau }}^{2}k_{p,\mathrm{air}}}\frac{{\left(\phi_{f}^{\max}\right)}^{3}}{{\left(1-\phi_{f}^{\max}\right)}^{2}}$$

## Appendix C. Analysis of Variance (ANOVA) for the Data of Air Permeability

In this appendix, analysis of variance (ANOVA) was used to analyze the differences among average values of the air permeability of baked-up anode samples to different baking temperatures. The data of air permeability (Figure 7) are listed in Table A1:

**Table A1:** The data of air permeability.

Temperature (°C)	25	200	300	400	500	600	700	1100
Air permeability (nPm)	0.217	0.440	0.655	0.895	0.416	0.733	0.126	0.862
	0.409	0.631	0.651	0.606	0.859	0.476	0.565	0.164
	0.192	0.689	1.017	1.105	0.738	0.718	1.370	0.450
	0.825	0.918	1.186	1.247	0.604	1.013	0.813	0.658
	0.350	0.572	0.289	0.804	1.186	1.144	0.752	
	0.620	0.884	1.389			0.973		

According to the number of chosen baking temperatures, the data of air permeability were divided into eight groups and the average value of each group was calculated, as shown in Table A2:

**Table A2:** Average value of each group data of air permeability.

Group No.	1	2	3	4	5	6	7	8
Temperature (°C)	25	200	300	400	500	600	700	1100
Group size $n_{i}$	6	6	6	5	5	6	5	4
Average value (nPm) $\bar{P_{i}}$	0.436	0.689	0.865	0.931	0.761	0.843	0.725	0.534

Let k = 8 and N = 43 denote, respectively, the number of groups and the total number of measured data. Thus, the numerator $df_{1}$ and denominator $df_{2}$ degrees of freedom are, respectively, calculated as:

(A9) $$\{\begin{array}{l} df_{1}=k-1=7\\ df_{2}=N-k=35 \end{array}$$

Let $P_{\mathrm{ij}}$ denote the j-th value of individual data point in the i-th group (j = 1…$n_{i}$) in Table A1. Using the data in Table A1, the average value of total data points $\bar{P}$ is calculated by:

(A10) $$\bar{P}=\frac{\sum_{i=1}^{k}\sum_{j=1}^{n_{i}}P_{\mathrm{ij}}}{N}=0.726$$

Using the data in Table A2 and the Equation (A10), the sum of squares between groups SSB is calculated by:

(A11) $$SSB=\sum_{i=1}^{k}n_{i}{\left({\bar{P}}_{i}-\bar{P}\right)}^{2}=1.077$$

where $n_{i}$ and ${\bar{P}}_{i}$ denote, respectively, the size and the average value of i-th group data of air permeability.

Using the data in Table A1 and Table A2, the residual variation SSE is calculated by:

(A12) $$SSE=\sum_{i=1}^{k}\sum_{j=1}^{n_{i}}{\left(P_{\mathrm{ij}}-{\bar{P}}_{i}\right)}^{2}=3.255$$

Using the data in Table A1 and the Equation (A10), the total sum of squares SST is calculated by:

(A13) $$SST=\sum_{i=1}^{k}\sum_{j=1}^{n_{i}}{\left(P_{\mathrm{ij}}-\bar{P}\right)}^{2}=4.332$$

Combining Equations (A11), (A12), and (A13), one can obtain:

(A14) SST=SSB+SSE

Using Equations (A9), (A11), and (A12), the test statistic F is then calculated, as follows:

(A15) $$F=\frac{SSB/df_{1}}{SSE/df_{2}}=1.654$$

When the level of significance equals α=10%, the critical value of F-distribution at α is:

(A16) $$F_{\alpha }\left(df_{1},df_{2}\right)=F_{0.1}\left(7,35\right)=1.896$$
